# Moving beyond animal models: enriched environments and human substance use disorders

**DOI:** 10.3389/fnbeh.2025.1629918

**Published:** 2025-09-05

**Authors:** Lila Barillot, Claudia Chauvet, Emeline Chauchard, Marc Besnier, Ghina Harika-Germaneau, Xavier Noël, Nemat Jaafari, Marcello Solinas, Armand Chatard

**Affiliations:** ^1^Université de Poitiers, Centre National de la Recherche Scientifique (CNRS) 7295, Centre de Recherches sur la Cognition et l’Apprentissage, Poitiers, France; ^2^Unité de Recherche Clinique, Centre Hospitalier Laborit, Poitiers, France; ^3^Laboratoire de Psychologie Médicale et d’Addictologie, Université Libre de Bruxelles, Brussels, Belgium; ^4^Université de Poitiers, Institut National de la Santé et de la Recherche Médicale (INSERM) U1084, Laboratoire de Neurosciences Expérimentales et Cliniques, Poitiers, France

**Keywords:** addiction, alcohol, tobacco, environmental aspects, relapse

## Abstract

**Background:**

Preclinical studies have shown that exposure to a multisensory, stimulating environment (environmental enrichment, EE) can prevent the development of addictive behaviors and reduce the risk of relapse in animal models. However, the extent to which these preclinical findings apply to human addiction remains largely unknown. In this research, we investigated the role of EE in human substance use disorders (SUDs).

**Methods:**

A new self-report measure of perceived EE was developed to test, in human participants, whether EE is associated with lower levels of SUD. This scale was administered to two distinct groups: regular smokers (*N* = 286) and patients diagnosed with severe alcohol use disorder (*N* = 52). Smokers also provided demographic information and data on nicotine use, while patients with alcohol use disorder reported pre-hospitalization drug intake, detoxification history, and levels of depression and anxiety.

**Results:**

The EE scale demonstrated adequate psychometric properties, including a stable factorial structure and high test–retest reliability over 1 month. Among smokers, higher scores were significantly associated with lower nicotine consumption, dependence and craving. In patients with alcohol use disorder, lower scores were linked to a history of more frequent relapse. These effects were independent from depression and anxiety.

**Conclusion:**

Environmental enrichment, as perceived and self-reported by individuals, appears to be a promising construct for understanding vulnerability and resilience in human addiction. The scale may serve as a valuable translational tool between preclinical and clinical models, with potential implications for the development of new intervention strategies for SUD.

## Introduction

Substance use disorder (SUD) is a multifaceted, chronic, and relapsing disorder that emerges from the interplay of biological, psychological, and social factors (Griffiths, 2005; Koob et al., 1998; Skewes and Gonzalez, 2013). The biopsychosocial model is the most influential approach to understanding how these different levels influence the development and maintenance of addictive behaviors (Donovan and Marlatt, 2007; Marlatt et al., 1988). Among these factors, the environment plays a key role, shaping vulnerability to SUD and affecting the likelihood of relapse after cessation (Ewald et al., 2019; Gordon, 2002; Sinha, 2007). To prevent SUD or relapse, it is necessary to identify the role of environmental protective and risk factors associated with drug use.

Extensive preclinical literature demonstrates that exposure to environmental enrichment (EE) reduces addictive behaviors and the neurobiological effects of drugs in rodents (Galaj et al., 2020; Malone et al., 2022; Solinas et al., 2010). In rats reared in EE, cocaine and amphetamine self-administration is reduced, as well as motivation for drug and propensity to relapse (Bardo et al., 2001; Green et al., 2002; Stairs et al., 2006). Regarding therapeutic effects of EE, in rats trained to self-administer cocaine it has been shown that 1 month of EE during abstinence substantially reduces cocaine seeking and cue- or stress-induced reinstatement (Chauvet et al., 2009). These results have been replicated for different models of addiction and drugs belonging to different pharmacological classes (Gauthier et al., 2017; Li et al., 2015; Maccioni et al., 2022; Sanchez et al., 2013; Sikora et al., 2018; Thiel et al., 2009). The protective and therapeutic effects of EE against addiction are believed to depend on neurophysiological mechanisms altering stress response and brain reactivity to cues (Chauvet et al., 2012; Crofton et al., 2015; Solinas et al., 2021). Importantly, EE has been shown to induce synaptic plasticity and neurogenesis in the hippocampus, which are thought to support new learning and improved memory (Solinas et al., 2010). These effects have important clinical and societal implications. Transposed to humans, EE approaches could enable the development of new therapeutic interventions aimed at enriching the environment of patients suffering from SUD. Therefore, understanding the link between EE and addiction in humans is critical to propose new therapeutic approaches for the treatment of SUD.

Enriched environments combine complex stimulation at different levels: motor stimuli (locomotion and physical activity), sensory stimuli (taste, olfactory, tactile, visual, auditory, and proprioceptive), novelty (exposure to relatively new stimulations), cognitive and affective stimuli (cognition, learning, and emotion), and social stimuli (social interaction) (Kempermann, 2019; Solinas et al., 2010). The aim of EE, beyond increasing the complexity of the environment, is to promote the wellbeing of individuals (Solinas et al., 2021; Thiel et al., 2009). Indeed, in animal models, EE offers a wide choice of naturally rewarding activities in which the individual engages voluntarily. EE exposes individuals to novel and challenging situations that can be solved, giving them a sense of control over their environment. Such stimulation has been shown to enhance brain plasticity, notably by promoting neurogenesis, synaptogenesis, and dendritic growth (van Praag et al., 2000; Sale et al., 2014).

The complexity of the environment and the diversity of stimulation are crucial features of EE, producing generalized effects such as arousal and learning (Kempermann, 2019), and collectively contributing to general wellbeing and stress reduction (Solinas et al., 2021). In this sense, EE is more than simply giving access to rewarding alternatives to drugs, as conceptualized by behavioral theories of choice (Acuff et al., 2019; Bickel and Vuchinich, 2000; Herrnstein, 1970). But how can we mimic EE in humans? What should be the components of an enriched environment?

Environmental enrichment in humans can be seen as an environment that allows multimodal and rewarding activities (Galaj et al., 2020), which stimulate physical, social and cognitive abilities while promoting wellbeing and fulfillment. For example, the practice of a regular physical activity has well-documented effects on cognitive functions, brain oxygenation and stress reduction (Salmon, 2001; Salzman et al., 2022). It could directly mirror voluntary physical exercise in animals (e.g., access to running wheels), which is a major contributor to neurogenesis, synaptic plasticity, and resilience to stress (Chieffi et al., 2017). The social dimension of EE involves group housing and social interactions between animals. These interactions are known to be highly rewarding and contribute to emotional wellbeing (Trezza et al., 2011). In humans, the availability and quality of supportive relationships strongly influence wellbeing, emotion regulation and treatment outcomes (Thoits, 2011), thus inducing comparable beneficial effects as in animals. In addition, EE provides a variety of positive sensory stimulations related to objects, sounds, textures, that promote arousal, exploration and brain plasticity (Kempermann, 2019). In humans, this may translate into the experience of a sensorially rich and diverse environment. Natural environments, for example, have been shown to foster wellbeing and reduce stress through multimodal sensory input (Franco et al., 2017). Mindfulness studies suggest that the subjective awareness and appraisal of such stimuli may partly mediate their psychological impact (Howell et al., 2011). Another key feature of EE is its ability to stimulate cognitive functions. In humans, cognitively stimulating activities could be mental tasks that require attention, focus, and information processing (Wilson et al., 2005, 2010), and may include cultural activities including watching stimulating content on television, or playing a musical instrument (Crowe et al., 2003; Hultsch et al., 1993; Verghese et al., 2003). These cultural and artistic activities have been linked to reduced cognitive decline and improved stress regulation (Fancourt and Steptoe, 2019; Verghese et al., 2003). Music perception involves brain networks related to learning, emotion and sensory processing (Vuust et al., 2022)—key mechanisms of EE in animals. Though culturally specific, these experiences may capture core components of cognitive enrichment such as novelty, learning and cognitive engagement. Finally, an important feature of EE is the active engagement of animals with their environment. EE promotes exploratory behaviors and motivation (Solinas et al., 2010). In humans, engaging in diverse and meaningful activities leads to better cognitive performances (Schaie, 1984). These effects may rely on overlapping mechanisms, such as increased motivational drive, curiosity, and exposure to novelty, reflecting the behavioral and neurobiological benefits of EE in animals.

Importantly, interventional studies have demonstrated that several of these EE factors play a positive role in SUD. Notably, physical activity (Haasova et al., 2013; Hallgren et al., 2017; Thompson et al., 2020; Wang et al., 2014), cognitive training (Czuchry and Dansereau, 2003; Rass et al., 2015; Rezapour et al., 2017; Rupp et al., 2012), and social support (Garmendia et al., 2008; Havassy et al., 1991; Jia et al., 2024) have shown promising effects. In particular, physical activity and cognitive training could reduce craving (Haasova et al., 2013; Rupp et al., 2012), and social support could reduce the risk of relapse for different types of drugs (Garmendia et al., 2008; Havassy et al., 1991). Mindfulness meditation can also help regulate stress and emotions (Barillot et al., 2023; Chiesa and Serretti, 2009; Fjorback et al., 2011; Guendelman et al., 2017), which could be key factors in the effectiveness of EE (Crofton et al., 2015; Solinas et al., 2010, 2021), while providing cognitive stimulation (Sampedro-Piquero et al., 2019).

To study the influence of EE on SUD in humans, it is important to measure the extent to which people’s daily environment feels “enriched,” or stimulant and link this measure to problematic drug use. Although EE in animals is defined through exposure to defined and objective environmental parameters (number toys, partners and running wheels, frequency of exposure to novelty, etc.), a study found that animals’ relative experience of enrichment is critical to drug vulnerability. Indeed, mice reared in EE and later exposed to standard housing show increased sensitivity to cocaine compared to mice reared in standard conditions throughout (Nader et al., 2012). These findings suggest that the effects of environmental conditions do not only depend on the absolute and objective level of enrichment, but also on the organism’s sensitivity, modulated by context and past exposure. In humans, this influence of context and past experience may be reflected in the subjective perception of the environment, which is likely to influence its psychological effects. Therefore, assessing perceived enrichment is critical to understand the effects of EE in a translational approach.

Various tools have been developed to assess aspects of the environment in relation to addiction. For example, self-report scales like the World Health Organization Quality of Life (WHOQOL) (The WHOQOL Group, 1998) evaluate quality of life, focusing on physical and psychological health, social relationships, and the living environment. These measures emphasize general satisfaction (e.g., safety, transport, and access to services) and overall wellbeing. These dimensions are important to understand the consequences of SUD, which is negatively correlated with quality of life (Bratu et al., 2023). However, they do not directly reflect the diversity and complexity of environmental stimulation as conceptualized in preclinical EE studies. In addition, measures like the Environmental Reward Observation Scale (EROS) (Armento and Hopko, 2007) and the Pleasant Events Schedule (PES) (MacPhillamy and Lewinsohn, 1982) have been developed to assess the presence of environmental rewards (alternatives to drugs) (Acuff et al., 2019). While these tools provide valuable insights into alternative reinforcers, they primarily focus on general satisfaction or very specific events. As highlighted by Acuff et al. (2019), such satisfaction-based measures may not encompass all reinforcing activities (e.g., school activities, arduous physical activities that provide a sense of accomplishment rather than immediate pleasure). The authors also underline the lack of consensus on measuring these alternative reinforcers in clinical and applied contexts, a significant limitation for transposing findings from preclinical EE studies to humans. Importantly, although satisfaction and wellbeing are generally a consequence of EE, they are conceptually distinct and EE cannot be reduced to this outcome. A tool designed to more directly assess the perceived complexity and multimodality of EE may serve as a valuable complement to existing measures, offering new opportunities to examine how environmental stimulation relates to both wellbeing and SUD.

Our research aims to address these gaps by developing a measure grounded in the concept of EE, focusing on multimodal, fulfilling types of activity in the environment rather than satisfaction with experiences alone. Given that EE, by definition, involves a variety of sensory, cognitive, social, and physical stimulations acting together to promote engagement and brain plasticity, a multidimensional approach appeared necessary to reflect this richness. We also aimed to create a brief and practical tool suitable for use in clinical settings, particularly with patients diagnosed with severe alcohol use disorder (SAUD), for whom long or redundant questionnaires can be discouraging. Therefore, we developed a new self-report scale—the Environmental Enrichment Scale (EES)—to assess perceived EE across several domains. The EES captures whether individuals feel immersed in a stimulating environment, not only from a global perspective but also through distinct yet concise dimensions: cognitive and cultural, physical, social, and sensory. Deriving insight from preclinical studies, we hypothesized that a stimulating environment would be associated with lower drug use, reduced craving, and decreased dependence severity and relapse risk among individuals with SUD.

## Study 1

We conducted a first online study on regular tobacco consumers, to measure the association between the perceived EE and different addiction variables. We hypothesized that an environment perceived as stimulating should be associated with lower tobacco consumption, lower nicotine dependence and lower craving.

### Method

#### Participants

Study 1 was conducted online, with 283 regular smokers (125 self-identified females, 129 males, and 29 missing data) recruited through the research platform Prolific^1^ and included measures previously published (Solinas et al., 2024). We used the inclusion criteria suggested on the Prolific platform to select the participants for this study. The inclusion criteria required participants to be regular smokers at the time of the study, defined as individuals who smoked at least five cigarettes per day over the past year, residents of the United Kingdom or the United States, native English speakers, and aged 35 or older (*M*_age_ = 48.9, SD = 9.97). This age criterion was chosen to oversample participants with a relatively long history of smoking. Preliminary analyses revealed no significant differences between participants from the two countries, allowing the data to be merged.

Participants completed the survey on 3 March 2022. Approximately 30 days later, on 4 April 2022, they were recontacted via Prolific and invited to complete a follow-up survey. This follow-up aimed to assess the test–retest reliability of the EES.

The study was approved by the local Ethics Committee. All participants provided informed consent before participating, were thoroughly debriefed, and received monetary compensation of £10 per hour. Table 1 summarizes the demographic characteristics of the sample.

**TABLE 1:** Descriptive statistics.

Variable	*N*	Missing	Mean	SD	Min	Max
**Initial assessment**						
Tobacco consumption	276	7	15.5	7.94	4.0	50.0
Craving	283	0	3.74	1.56	1.01	6.99
Nicotine dependence	283	0	4.4	1.99	0.0	10.0
EES	283	0	4.14	1.06	1.04	6.67
**Follow-up**						
EES	283	0	4.16	1.06	1.71	6.52

Tobacco consumption refers to the self-reported number of cigarettes consumed per day at the time of the study. Craving was assessed using the QSU-brief, and nicotine dependence was measured with the Fagerström Test. EES stands for the Environment Enrichment Scale (see text for details). The follow-up was conducted 1 month later.

#### Measures

Participants reported the number of cigarettes they usually consume on a daily basis and completed three questionnaires: the EES, a measure of craving and a measure of nicotine dependence, as described in turn hereafter.

#### Cigarette consumption

Participants were asked to report the quantity of cigarettes they typically smoke daily. This was done to confirm their eligibility for the study and to provide a precise estimate of their cigarette consumption at the time of the study.

#### Environmental enrichment scale

Participants assessed the extent to which they perceived their surroundings as stimulating and enriching using the EES, a 13-item self-report instrument. The EES consists of five factors, each reflecting a different dimension of EE: Physical Activity (two items), Social Relationships (two items), Multisensory Immersion (two items), Cultural and Artistic Activities (two items), and Passion and Daily Engagement (five items). These dimensions and corresponding items were derived from literature on EE in animal models and relevant human research. The full list of items is presented in Table 2. Each item was rated on a 7-point Likert scale ranging from 1 (strongly disagree) to 7 (strongly agree).

**TABLE 2:** List of items and factor structure of the EES.

	Factor				
Items	1	2	3	4	5
My surroundings are full of rich sensations and various kinds of stimulation				0.69	
Often, I tell myself that my life is not very exciting			−0.57		
I engage in many physical and/or sports activities	0.92				
My physical and/or sports activities are a source of personal fulfillment	0.91				
I have a rich inner life			0.49		
I play and/or listen to a lot of music					0.41
I watch many stimulating movies and documentaries					0.47
I have many friends and social connections		0.91			
My social relationships are rich and stimulating		0.72			
I engage in a wide variety of diverse activities			0.30		
I have a passion in life (e.g., a hobby)			0.70		
I am surrounded by a stimulating environment				0.82	
I feel like my daily environment is dull, always the same			−0.59		
% variance	14.49	14.71	15.61	12.93	4.57

Entries are factor loadings in an explorative factor analysis. A principal axis factoring extraction method was used in combination with an oblimin rotation. Loadings below 0.3 are not depicted. The five factors are (1) Physical Activity, (2) Social Relationships, (3) Passion and Daily Engagement, (4) Multisensory Immersion, and (5) Cultural and Artistic Activities.

The EES was developed and refined over a 3-year period using convenience samples (e.g., students) before being applied and further tested in the present study. An exploratory factor analysis (EFA) was conducted to assess the scale’s factorial structure in our sample of regular smokers, followed by a confirmatory factor analysis (CFA) on follow-up data to validate this structure.

The EFA indicated a good model fit, with a Root Mean Square Error of Approximation (RMSEA) of 0.05 (90% CI: 0.01–0.06), a Tucker–Lewis Index (TLI) of 0.96, and a Chi-square test result of *p* = 0.015. These results suggest that the model provides a reasonably good representation of the data’s underlying structure. The five factors cumulatively explained 62.3% of the total variance (see Table 2). The decision to retain five factors was informed by parallel analyses conducted during the scale’s development phase, using student convenience samples. This method, which compares observed eigenvalues to those generated from random data, consistently supported a five-factor solution across samples.

All five factors were moderately correlated with each other (*r* between 0.28 and 0.62, *p* < 0.001), except for Cultural and Artistic Activity, which was not significantly correlated with the others (see Supplementary Table 1). We nonetheless chose to retain this factor, as it reflects a central source of stimulation frequently reported by participants during the development phase—particularly references to music, cinema (e.g., Netflix), and social media as meaningful forms of enrichment. Retaining this facet aligns with our theoretical objective of capturing the multidimensional nature of EE, as discussed earlier.

In the follow-up CFA conducted 1 month later, the model fit remained strong, with a CFI of 0.93 and a TLI of 0.91, indicating a good fit. The RMSEA of 0.08 suggested a moderate but acceptable fit, consistent with the scale’s multidimensional structure. Although the EES was designed to capture distinct yet complementary forms of environmental stimulation—reflecting the theoretical foundation of EE as a multi-sensory and multi-domain construct—our primary interest was in the global level of perceived stimulation, rather than in the predictive value of each factor considered independently. This justifies the use of a total score in the main analyses. The significant Chi-square (*p* < 0.001) was expected, given its sensitivity to sample size; therefore, more emphasis was placed on other fit indices such as RMSEA, CFI, and TLI.

For the analyses, a total score was calculated by averaging the 13 items of the EES. This approach provides a comprehensive measure of participants’ perceptions of their environment as enriching and stimulating, while still respecting the scale’s multidimensional theoretical foundation. Although we focus here on the total EE score, the specific associations between each factor and the main outcomes are reported in Supplementary material.

The internal reliability of the EES was excellent, with a Cronbach’s alpha of 0.884 at the initial measurement and 0.875 at the 1-month follow-up. These values indicate a high level of internal consistency across the 13 items. Furthermore, test–retest reliability over the 1-month interval was strong, with an intraclass correlation coefficient of 0.859 (*F* = 13.2, *p* < 0.001), demonstrating good temporal stability of the scale. These results provide additional evidence for the robustness of the EES as a psychometrically sound instrument for assessing perceived EE.

#### Craving questionnaire

The Brief Questionnaire of Smoking Urges (QSU-brief; Cox et al., 2001) was administered at Time 1 to assess cigarette craving. This 10-item instrument measures the immediate desire to smoke (e.g., “All I want right now is a cigarette” and “I have an urge for a cigarette”) and has been shown to predict drug-seeking behaviors (Solinas et al., 2024). Participants rated each item on a 7-point Likert scale ranging from 1 (strongly disagree) to 7 (strongly agree). In the present sample, internal consistency was excellent (Cronbach’s alpha = 0.95). Responses to the 10 items were summed to generate a composite craving score.

#### Nicotine dependence

At Time 1, participants completed the Fagerström Test for Nicotine Dependence (Heatherton et al., 1991), a widely used instrument to assess the severity of nicotine addiction. This measure provides an ordinal score of nicotine dependence related to cigarette smoking. The questionnaire consists of six items that assess cigarette consumption, compulsion to smoke, and dependence (e.g., “Do you smoke even when you are so ill that you are in bed most of the day?”). The six items were summed to yield a total score ranging from 0 to 10.

#### Statistical analyses

Given the presence of outliers in the data, robust statistical methods were employed (Wilcox, 2013). Specifically, Spearman’s rank-order correlation, a non-parametric approach, was used to ensure the validity of the analyses. All analyses were performed using R version 3.6.1 (R Core Team, 2021).

### Results

Table 1 presents the means and standard deviations of the different variables used in this study. One participant was excluded from the main analyses because she did not meet our inclusion criterion of consuming at least five cigarettes per day at the time of the study (see Table 1).

The correlations between the different variables are shown in Table 3. As expected, tobacco consumption was positively associated with tobacco craving at the time of the study, as well as with participants’ levels of nicotine dependence in the Fagerström questionnaire. Additionally, participants with more severe nicotine dependence were more likely to report higher cravings at the time of the study.

**TABLE 3:** Correlations between environmental enrichment, tobacco consumption, craving, and nicotine dependence.

Variables	Statistics	Tobacco consumption	Craving	Nicotine dependence	Environmental enrichment (initial)	Environmental enrichment (follow-up)
Tobacco consumption	Spearman’s rho	–				
	*p*-Value	–				
Craving	Spearman’s rho	0.204	–			
	*p*-Value	< 0.001	–			
Nicotine dependence	Spearman’s rho	0.704	0.329	–		
	*p*-Value	< 0.001	< 0.001	–		
Environmental enrichment (initial)	Spearman’s rho	−0.134	−0.125	−0.185	–	
	*p*-Value	0.026	0.035	0.002	–	
Environmental enrichment (follow-up)	Spearman’s rho	−0.076	−0.053	−0.128	0.856	–
	*p*-Value	0.210	0.371	0.032	< 0.001	–

Tobacco consumption refers to the self-reported number of cigarettes consumed per day at the time of the study. Craving was assessed using the QSU-brief, and nicotine dependence was measured with the Fagerström Test. EES stands for the Environment Enrichment Scale (see text for details). The follow-up was conducted 1 month later.

**FIGURE 1:**
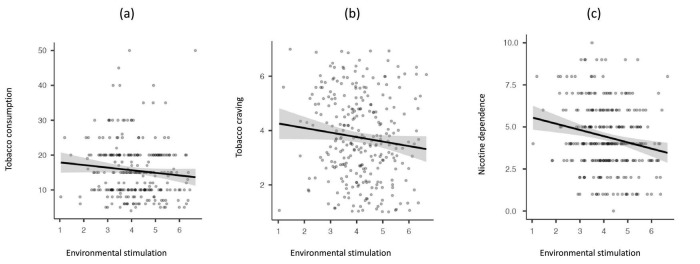
Scatter plots showing the rank-order correlation (with 95% CI) between environmental enrichment (initial assessment) and tobacco consumption **(a)**, craving **(b)**, and nicotine dependence **(c)**.

As shown in Table 3, the EES was negatively associated with tobacco consumption, tobacco craving, and nicotine dependence. At the initial assessment, the correlations between the EES and both tobacco consumption and craving were relatively small and only marginally significant. However, the correlation between EES and nicotine dependence was stronger and more significant (see Figure 1).

The EES measured after 1 month was not associated with tobacco consumption or craving assessed 1 month earlier. However, the EES at follow-up was negatively correlated with nicotine dependence assessed 1 month earlier.

### Discussion of study 1

The results of this first study provide preliminary evidence supporting the role of perceived EE in tobacco use and dependence. As expected, participants with higher scores on the EES reported lower levels of nicotine dependence, and reduced tobacco consumption and craving. This suggests that individuals who perceive their environment as more stimulating and enriched tend to show lower levels of tobacco-related problems.

While the correlations between the EES and both cigarette consumption and craving were small, the association between the EES and nicotine dependence was larger. This pattern supports the idea that perceived EE may be more closely related to the structural features of addiction, such as dependence, than to more situational or transient aspects, such as state craving. Interestingly, this finding held even when using EES scores collected 1 month after the initial assessment, which still significantly correlated with nicotine dependence reported 1 month earlier. This temporal association, although cross-sectional, provides some support for the robustness and stability of the link between EE and addiction severity.

These findings are consistent with the animal literature on EE, which has repeatedly shown that enriched environments can reduce drug-seeking behaviors and vulnerability to addiction (Malone et al., 2022; Stairs and Bardo, 2009) and notably nicotine addiction (Sikora et al., 2018). Translating this to human behavior, our results suggest that individuals who perceive their daily lives as richer in stimulation—be it social, physical, sensory, or cognitive—may be less prone to developing entrenched patterns of dependence.

In sum, this first study provides encouraging evidence that perceived EE is meaningfully related to tobacco addiction severity in regular smokers. Although further research is needed to clarify the nature of this relationship and the mechanisms behind it, these results highlight the potential value of perceived environmental stimulation as a psychological correlate of addictive behavior.

## Study 2

Building on the results of Study 1, which indicated a negative association between perceived EE and tobacco addiction severity, Study 2 aimed to examine whether similar patterns would emerge in a different population—individuals diagnosed with another SUD and actively seeking treatment. Specifically, we investigated whether perceived EE was associated with relapse risk in patients undergoing detoxification for SAUD, a clinically more complex and severe condition.

### Method

#### Participants

The study included 52 patients treated at the Addiction Clinical Unit of Henri Laborit Psychiatric Hospital, located in a midsize city in France, between January 2020 and September 2020. All participants were diagnosed with SAUD based on DSM-5 criteria (American Psychiatric Association, 2013). A trained psychiatrist confirmed the diagnosis using the Mini International Neuropsychiatric Interview (MINI; Sheehan et al., 1998). SAUD was defined as the presence of six or more criteria, in accordance with DSM-5 guidelines. The study received approval from the local Ethics Committee, and all patients provided written informed consent. The demographic characteristics of the sample are summarized in Table 4.

**TABLE 4:** Demographic, addiction-related, and clinical characteristics of the sample.

Variable	%	Mean	SD	Min	Max
**Demographic variables**					
Age	–	45.08	12.11	24	71
Gender (% female)	28.8	–	–	–	–
Nationality (% French)	94.2	–	–	–	–
Mother tongue (% French)	98.1	–	–	–	–
Highest diploma (mi*n* = 1, max = 7)	–	3.39	1.35	1	7
Currently employed (% yes)	44.2	–	–	–	–
Marital status (% single)	46.2	–	–	–	–
Children (% yes)	55.8	–	–	–	–
**Addiction-related variables**					
Other addictions (% none)	1.9	–	–	–	–
Age of first alcohol use	–	16.59	9.11	6	56
Age of dependence onset	–	31.78	13.69	14	16
Number of prior alcohol detoxifications (relapse)	–	2.22	4.82	0	30
Total alcohol consumption (TAC)	–	221.24	136.33	30	600
**Clinical variables**					
Depression (BDI)	–	23.67	6.81	14	44
Anxiety (STAI)		40.88	15.63	20	76

TAC = total alcohol consumption, representing the average daily alcohol consumption just before entering treatment. STAI = State-Trait Anxiety Inventory, used to measure state anxiety levels in the present study. BDI = Beck Depression Inventory, used to assess the severity of depression at the time of the study.

#### Measures

During their hospitalization, participants completed a survey that included all measures relevant to this study. The survey included measures of current and past alcohol consumption (see Table 4), as well as the EES used in the previous study, a measure of depression, and a measure of state anxiety.

#### Pre-hospitalization drug intake

Participants reported the type of alcohol they consumed and their daily alcohol intake just before hospitalization. The psychiatrist then converted these data into a total alcohol consumption (TAC) score by calculating the average daily intake of pure alcohol in grams. This was done by multiplying the volume of each type of alcohol consumed by its alcohol by volume (ABV) percentage and the density of ethanol (0.789 g/ml), yielding the total grams of pure alcohol consumed per day just before entering treatment. This served as the primary measure of drug consumption in the present study.

#### Detoxification history

Participants reported the number of detoxification treatments they had undergone throughout their lifetime. This served as the primary measure of relapse in the present study.

#### Environmental enrichment scale

A French version of the EES was used to assess participants. Consistent with the previous study, we tested the five-factor structure of the scale using CFA. The model fit statistics suggest a less-than-ideal fit (χ^2^(43) = 68.7, *p* = 0.007, CFI = 0.85, TLI = 0.77, SRMR = 0.097, RMSEA = 0.108), but given the small sample size and clinical nature of the participants, this outcome may not be unexpected. Despite this, the scale demonstrated good internal reliability, with Cronbach’s alpha at α = 0.75. As a result, we averaged the 13 items to form a composite score of EE.

#### Depression

The Beck Depression Inventory (BDI) was used to assess the severity of depressive symptoms in participants. The BDI is a widely used self-report scale consisting of 21 items that measure various cognitive, emotional, and physical symptoms associated with depression. Each item is rated on a 4-point scale, with higher scores indicating more severe depressive symptoms. In this study, the average score was 23.67 (see Table 4). These relatively high scores are not surprising, given the clinical nature of the present sample.

#### Anxiety

Anxiety was measured using the State version of the State-Trait Anxiety Inventory (STAI), a widely used self-report instrument designed to assess the current intensity of anxiety symptoms. The STAI-State version consists of 20 items, each rated on a 4-point scale, with higher scores indicating higher levels of state anxiety (temporary and situational anxiety). In this study, the average score was 40.88 (Table 4). These scores suggest a moderate to high level of situational anxiety among participants.

#### Statistical analyses

Due to the presence of outliers in the data, Spearman’s rank-order correlation, which is robust to outliers, was used to ensure the validity of the analyses. All analyses were performed using R version 3.6.1 (R Core Team, 2021).

### Results

First, bivariate correlations were conducted to examine whether individual differences in EE score were related to addiction-related and clinical variables in this sample. We then tested whether the relationship between EE score and addiction-related variables remained significant after controlling for STAI and BDI scores.

The bivariate correlations are presented in Table 5. As shown, individual differences in EES were not significantly associated with TAC. EES was negatively associated with depression and anxiety, though these correlations did not reach conventional significance levels. Depression, but not state anxiety, was positively related to TAC and relapse, although these relationships were also not significant. However, as expected, patients who perceived their environment as less stimulating were significantly more likely to relapse as measured by the number of previous detoxifications.

**TABLE 5:** Correlations between environmental enrichment, total alcohol consumption, relapse rate, depression, and anxiety.

Variables	Statistics	EES	TAC	Relapse	BDI	STAI
EES	Spearman’s rho	–				
	*p*-Value	–				
TAC	Spearman’s rho	−0.005	–			
	*p*-Value	0.973	–			
Relapse	Spearman’s rho	−0.309	0.150	–		
	*p*-Value	0.029	0.297	–		
BDI	Spearman’s rho	−0.246	0.210	0.114	–	
	*p*-Value	0.078	0.136	0.432	–	
STAI	Spearman’s rho	−0.176	0.078	−0.008	0.714	–
	*p*-Value	0.213	0.580	0.956	< 0.001	–

Interestingly, the correlation between EES and relapse history remained significant after controlling for depression and anxiety (see Table 6). This suggests that EE has a specific effect on relapse rates, independent of clinical variables. Similarly, partial correlation analyses showed that the relationship between EES and relapse is independent of participants’ age (*r*_s_ = −0.287, *p* = 0.045), age of first alcohol use (*r*_s_ = −0.307, *p* = 0.037), and age of dependence onset (*r*_s_ = −0.331, *p* = 0.033). This further indicates that EES has a distinct effect on relapse rates, beyond age-related variables.

**TABLE 6:** Partial correlations between EES and addiction-related and clinical variables.

Variables	Statistics	EES	Relapse	TAC
EES	Spearman’s rho	–		
	*p*-Value	–		
Relapse	Spearman’s rho	−0.296	–	
	*p*-Value	0.041	–	
TAC	Spearman’s rho	0.050	0.119	–
	*p*-Value	0.731	0.420	–

Controlling for “BDI” and “STAI.”

### Discussion of study 2

The results of Study 2 suggest that perceived EE is negatively associated with relapse history among patients treated for SAUD. This association remained significant even after controlling for clinical variables such as depression, anxiety, and age-related factors, supporting the idea that EE may independently contribute to relapse vulnerability. However, given the cross-sectional and correlational nature of the data, no firm conclusions can be drawn about the direction of the association. It remains possible that low EE increases relapse vulnerability, or conversely, that repeated relapses reduce access to or engagement in enriching experiences and general perception of EE. Regardless of the causal direction, the present findings establish a meaningful link between perceived enrichment and addiction-related outcomes—a critical first step in translating preclinical models of environmental stimulation to human populations.

In contrast to Study 1, where EES scores were negatively associated with tobacco consumption, no association was found here between EES scores and TAC. This discrepancy may reflect differences in population and context: while Study 1 involved non-treatment-seeking smokers, participants in Study 2 were hospitalized and actively engaged in treatment. It is also possible that the effect of EE is more closely related to relapse risk than to levels of substance use *per se*—a pattern consistent with preclinical findings in which EE reduces relapse independently of prior drug intake (Chauvet et al., 2009; Sikora et al., 2018; Thiel et al., 2009).

Taken together, these findings extend those from Study 1 and provide additional support for the relevance of environmental stimulation in understanding addiction-related outcomes.

## General discussion

While several studies have identified risk factors for addiction (Brook et al., 2015; Hägele et al., 2014; Nawi et al., 2021), protective factors remain less understood. To address this gap, we developed the EES, a self-report instrument grounded in the concept of EE, extensively validated in animal models (Galaj et al., 2020; Laviola et al., 2008). The EES demonstrated good psychometric properties and excellent 1-month test–retest reliability.

Consistent with our hypotheses and the preclinical literature, higher perceived EE was associated with lower levels of nicotine dependence, craving, and tobacco consumption among smokers, and with a lower likelihood of relapse in patients undergoing treatment for SAUD. These findings support the view that perceived environmental stimulation is a meaningful protective correlate of addiction severity and relapse vulnerability (Solinas et al., 2021; Sikora et al., 2018).

Although some of the associations observed in our studies—such as the correlation between EES scores and relapse in smokers—were modest in magnitude, this is consistent with theoretical expectations. EE is often conceptualized not as a direct suppressor of addictive behaviors, but rather as a protective buffer that may mitigate vulnerability in the face of triggers such as stress, craving, or environmental cues (Chauvet et al., 2012). In this framework, high levels of perceived EE are expected to play a moderating role, reducing the likelihood of relapse under high-risk conditions. Therefore, even small-to-moderate effect sizes can be clinically meaningful, particularly if EE is leveraged to enhance resilience within intervention programs.

In animal models, EE reduces nicotine sensitization, modulates neurobiological responses (Malone et al., 2022; Stairs and Bardo, 2009), and decreases relapse rates (Galaj et al., 2020; Sikora et al., 2018), probably by reducing stress (Crofton et al., 2015; Galaj et al., 2020; Solinas et al., 2010) and enhancing access to alternative sources of reward. Similar mechanisms may underlie the associations observed here. In the context of alcohol addiction, our results also align with rodent studies linking EE to reduced relapse (Campbell et al., 2019), potentially via improved stress regulation (Crofton et al., 2015; Galaj et al., 2020; Pang et al., 2019; Solinas et al., 2010). Various human interventions mirror these effects. Physical activity (Manthou et al., 2016; Cabé et al., 2021), social support (Dobkin et al., 2002; Longabaugh et al., 1993; Stevens et al., 2015), mindfulness, and cognitive training (Garland et al., 2010, 2014; Rupp et al., 2012; Czuchry and Dansereau, 2003) have all been linked to reduced craving, consumption, and relapse, suggesting that stimulating environments can promote resilience. Furthermore, physical activity and social support produce biological effects in humans that are consistent with those observed in EE animals. For instance, regular aerobic exercise in humans has been shown to increase peripheral levels of brain-derived neurotrophic factor (Huang et al., 2014) and to lower sympathetic nervous system and hypothalamic-pituitary-adrenal (HPA) axis reactivity (Anderson and Shivakumar, 2013). Similarly, social support has been associated with reduced stress responses, potentially via modulation of HPA axis activity (DeVries et al., 2003).

Differently from what we found tobacco smokers, the EES was not significantly associated with TAC in Study 2. This may be due to the retrospective nature of the TAC measure, which can induce under-reporting or recall errors (Del Boca and Darkes, 2003; Livingston and Callinan, 2015), or to the inconsistent and paradigm-dependent effects of EE on alcohol intake observed in animal studies (e.g., Bahi and Dreyer, 2020; Deehan et al., 2007, 2011; Ellison, 1981; Rockman et al., 1988). In humans, biological and social factors—such as peer influence—may limit or counteract the potential protective effect of EE on consumption (Acuff et al., 2019; Enoch, 2011; Schuckit, 2009; Thomasson, 2002; Borsari and Carey, 2001; Edwards et al., 2015; Larsen et al., 2009).

Unexpectedly, EE scores were also not significantly linked to depression or anxiety symptoms. This diverges from preclinical results (Laviola et al., 2008) and from human studies showing protective effects of environmental stimulation on mood (Doré et al., 2016; Miller et al., 2019; Félix et al., 2024). In our clinical sample, this could reflect ceiling effects, withdrawal fluctuations, or neurobiological disruptions linked to SAUD (Boden and Fergusson, 2011; Kushner et al., 2000; Brown et al., 1991; Driessen et al., 2001).

### Limitations and future directions

Several limitations should be acknowledged in this research. First, Study 1 did not include the time elapsed since the last cigarette, a key variable influencing craving (Chandra et al., 2011; Tiffany and Drobes, 1991). Second, Study 2 relied on a smaller, clinical sample and cross-sectional data. The use of past detoxifications as a proxy for relapse may have introduced imprecision, as not all relapses lead to hospitalization. Additionally, comorbidities, cognitive impairment, or concurrent substance use—frequent in clinical populations—were not assessed (Bernardin et al., 2014; Di Nicola et al., 2023; Ramey and Regier, 2019; Weinberger et al., 2017), potentially confounding the observed associations. The direction of the relationship also remains unclear: while low EE may increase addiction risk, addiction may itself reduce environmental stimulation via social withdrawal or lifestyle changes (Arabaci et al., 2020; Lander et al., 2013; Wiers and Verschure, 2021). Thus, environmental depletion may be a consequence, rather than a cause, of addiction severity. It is therefore essential to consider the potentially bidirectional relationship between environmental stimulation and addictive behaviors.

Limitations also arise from the nature of the EES as a declarative self-report measure. Like other introspective tools, it depends on participants’ self-awareness and can be biased by personality traits or recent changes in perception (Eisenhower et al., 1991; Shiffman et al., 1997; Zuckerman, 2006). Indeed, behavioral changes can precede and be independent from cognitive awareness (Acuff et al., 2019). Moreover, perceived EE may not always reflect objective environmental stimulation. Interestingly, animal studies show that perceived loss of enrichment, rather than its absolute level, increases drug vulnerability (Nader et al., 2012). These considerations open valuable avenues for future research: combining subjective and objective measures of EE, assessing changes across stages of addiction and recovery, and testing targeted interventions in clinical settings. For instance, the EES could be repeated at different time points to monitor changes and provide insights into the dynamic interplay between environmental stimulation and substance use.

Finally, while the present studies provide evidence for the structural, temporal, and predictive validity of the EES, they offer only preliminary insights into its discriminant validity. In Study 2, the EES predicted addiction-related outcomes beyond depression and anxiety, but future research should explore its convergent and discriminant validity more thoroughly. This includes examining its relationships with constructs such as social support, perceived stress, or personality traits (e.g., openness to experience). Establishing these connections would help better situate the EES within the broader nomological network of psychological constructs.

## Clinical implications and conclusion

Despite these limitations, our findings underscore the potential value of EE as a modifiable therapeutic target in the treatment and prevention of SUD. Interventions designed to increase environmental stimulation—through accessible, multimodal activities—may help reduce dependence severity and relapse vulnerability (Barillot et al., 2023; Solinas et al., 2021). For instance, a recent randomized controlled trial is testing an EE intervention in patients with AUD, using physical activity combined with cognitive games, and mindfulness practice in multisensory virtual environments (Barillot et al., 2023). The EES could support these efforts as a brief, multidimensional tool to assess environmental stimulation in applied settings.

In conclusion, perceived EE appears meaningfully associated with key addiction-related outcomes. By bridging preclinical insights with human data, this research contributes to a better understanding of environmental factors in addiction and opens new perspectives for prevention and care.

## Data Availability

The datasets presented in this study can be found in online repositories. The names of the repository/repositories and accession number(s) can be found in the article/Supplementary material. The anonymized data file and code for the present studies are available at: https://osf.io/yjumw/.

## Footnotes

1
https://www.prolific.com/
