# Supplementary material for: Moving beyond animal models: enriched environments and human substance use disorders
Source: Front Behav Neurosci. 2025 Sep 5;19:1629918. doi: 10.3389/fnbeh.2025.1629918 (PMC12446247; doi:10.3389/fnbeh.2025.1629918)
Supplement: Supplementary file 1 [file Data_Sheet_1.docx]

**Supplementary Online Material**

The tables presented below are intended to complement the manuscript titled "Beyond Animal Models: The Association of Enriched Environments with Human Addictions." Each table is referenced in the main text of the manuscript as a supplementary table (Table S) and is listed in the order in which it appears. Unless specified otherwise, the first measurement of the Environmental Stimulation Scale is used in the analysis (see text).

- Table S1 presents the correlations between the different factors of the Environmental Stimulation Scale (ESS).
- Table S2 provides the correlations between each factor of the ESS and cigarette consumption.
- Table S3 details the correlations between each factor of the ESS and craving.
- Table S4 outlines the correlations between each factor of the ESS and nicotine dependency.

Table S1. Correlations between the different factors of the Environmental Stimulation Scale (ESS).

|  | | | | | | | | | | | | | |
| --- | --- | --- | --- | --- | --- | --- | --- | --- | --- | --- | --- | --- | --- |
|  |  |  |  |  |  |  |  |  |  |  |  |  |  |
|  | |  | | Physical  Activity | | Social  Relationship | | Passion and  Daily Engagement | | Multisensory  Immersion | | Artistic  Activity | |
| Physical Activity |  | Spearman's rho |  | — |  |  |  |  |  |  |  |  |  |
|  |  | p-value |  | — |  |  |  |  |  |  |  |  |  |
| Social Relationship |  | Spearman's rho |  | 0.439 |  | — |  |  |  |  |  |  |  |
|  |  | p-value |  | < .001 |  | — |  |  |  |  |  |  |  |
| Passion and Daily Engagement |  | Spearman's rho |  | 0.387 |  | 0.625 |  | — |  |  |  |  |  |
|  |  | p-value |  | < .001 |  | < .001 |  | — |  |  |  |  |  |
| Multisensory Immersion |  | Spearman's rho |  | 0.280 |  | 0.604 |  | 0.457 |  | — |  |  |  |
|  |  | p-value |  | < .001 |  | < .001 |  | < .001 |  | — |  |  |  |
| Artistic Activity |  | Spearman's rho |  | 0.009 |  | -0.060 |  | -0.065 |  | -0.058 |  | — |  |
|  |  | p-value |  | 0.883 |  | 0.312 |  | 0.274 |  | 0.331 |  | — |  |
|  | | | | | | | | | | | | | |

Note. Entries are rho coefficients. Each factor corresponds to the mean of the items that loaded onto the respective factor in the exploratory factor analysis (see Table 2 in the text), excluding loadings below the threshold of 0.30. For example, Physical Activity is the mean of items 3 and 4. For the third factor, Passion and Daily Engagement, items 2 and 13, which had negative loadings, were reverse scored before being averaged with items 5, 10, and 11.

Table S2. Correlations between each factor of the Environmental Stimulation Scale and cigarette consumption

|  | | | | | | | | | | | | | | | |
| --- | --- | --- | --- | --- | --- | --- | --- | --- | --- | --- | --- | --- | --- | --- | --- |
|  |  |  |  |  |  |  |  |  |  |  |  |  |  |  |  |
|  | |  | | Physical  Activity | | Social  Relationship | | Passion and  Daily Engagement | | Multisensory  Immersion | | Artistic  Activity | | Tobacco  consumption | |
| Physical  Activity |  | Spearman's rho |  | — |  |  |  |  |  |  |  |  |  |  |  |
|  |  | p-value |  | — |  |  |  |  |  |  |  |  |  |  |  |
| Social  Relationship |  | Spearman's rho |  | 0.439 |  | — |  |  |  |  |  |  |  |  |  |
|  |  | p-value |  | < .001 |  | — |  |  |  |  |  |  |  |  |  |
| Passion and  Daily Engagement |  | Spearman's rho |  | 0.387 |  | 0.625 |  | — |  |  |  |  |  |  |  |
|  |  | p-value |  | < .001 |  | < .001 |  | — |  |  |  |  |  |  |  |
| Multisensory  Immersion |  | Spearman's rho |  | 0.280 |  | 0.604 |  | 0.457 |  | — |  |  |  |  |  |
|  |  | p-value |  | < .001 |  | < .001 |  | < .001 |  | — |  |  |  |  |  |
| Artistic  Activity |  | Spearman's rho |  | 0.009 |  | -0.060 |  | -0.065 |  | -0.058 |  | — |  |  |  |
|  |  | p-value |  | 0.883 |  | 0.312 |  | 0.274 |  | 0.331 |  | — |  |  |  |
| Tobacco consumption |  | Spearman's rho |  | -0.218 |  | -0.101 |  | -0.167 |  | -0.093 |  | 0.172 |  | — |  |
|  |  | p-value |  | < .001 |  | 0.093 |  | 0.005 |  | 0.125 |  | 0.004 |  | — |  |
|  | | | | | | | | | | | | | | | |

Note. The entries are rho coefficients. As shown in the table, all factors are negatively correlated with tobacco consumption, except for the last one. The negative correlations for Physical Activity and Passion and Daily Engagement were significant at p < .05.

Table S3. Correlations between each factor of the Environmental Stimulation Scale and craving

|  | | | | | | | | | | | | | | | |
| --- | --- | --- | --- | --- | --- | --- | --- | --- | --- | --- | --- | --- | --- | --- | --- |
|  |  |  |  |  |  |  |  |  |  |  |  |  |  |  |  |
|  | |  | | Physical  Activity | | Social  Relationship | | Passion and  Daily Engagement | | Multisensory  Immersion | | Artistic  Activity | | Craving | |
| Physical  Activity |  | Spearman's rho |  | — |  |  |  |  |  |  |  |  |  |  |  |
|  |  | p-value |  | — |  |  |  |  |  |  |  |  |  |  |  |
| Social  Relationship |  | Spearman's rho |  | 0.439 |  | — |  |  |  |  |  |  |  |  |  |
|  |  | p-value |  | < .001 |  | — |  |  |  |  |  |  |  |  |  |
| Passion and  Daily Engagement |  | Spearman's rho |  | 0.387 |  | 0.625 |  | — |  |  |  |  |  |  |  |
|  |  | p-value |  | < .001 |  | < .001 |  | — |  |  |  |  |  |  |  |
| Multisensory  Immersion |  | Spearman's rho |  | 0.280 |  | 0.604 |  | 0.457 |  | — |  |  |  |  |  |
|  |  | p-value |  | < .001 |  | < .001 |  | < .001 |  | — |  |  |  |  |  |
| Artistic  Activity |  | Spearman's rho |  | 0.009 |  | -0.060 |  | -0.065 |  | -0.058 |  | — |  |  |  |
|  |  | p-value |  | 0.883 |  | 0.312 |  | 0.274 |  | 0.331 |  | — |  |  |  |
| Craving |  | Spearman's rho |  | -0.008 |  | -0.127 |  | -0.129 |  | -0.102 |  | 0.939 |  | — |  |
|  |  | p-value |  | 0.891 |  | 0.033 |  | 0.031 |  | 0.088 |  | < .001 |  | — |  |
|  | | | | | | | | | | | | | | | |

Note. The entries are rho coefficients. As shown in the table, most factors, including Social Relationship, Passion and Daily Engagement, and Multisensory Immersion, are negatively correlated with craving. In contrast, Physical Activity and Artistic Activity do not show a negative correlation. The negative correlations for Social Relationship and Passion and Daily Engagement were significant at p < .05.

Table S4. Correlations between each factor of the Environmental Stimulation Scale and nicotine dependency

|  | | | | | | | | | | | | | | | |
| --- | --- | --- | --- | --- | --- | --- | --- | --- | --- | --- | --- | --- | --- | --- | --- |
|  |  |  |  |  |  |  |  |  |  |  |  |  |  |  |  |
|  | |  | | Physical  Activity | | Social  Relationship | | Passion and  Daily Engagement | | Multisensory  Immersion | | Artistic  Activity | | Nicotine  Dependency | |
| Physical  Activity |  | Spearman's rho |  | — |  |  |  |  |  |  |  |  |  |  |  |
|  |  | p-value |  | — |  |  |  |  |  |  |  |  |  |  |  |
| Social  Relationship |  | Spearman's rho |  | 0.439 |  | — |  |  |  |  |  |  |  |  |  |
|  |  | p-value |  | < .001 |  | — |  |  |  |  |  |  |  |  |  |
| Passion and  Daily Engagement |  | Spearman's rho |  | 0.387 |  | 0.625 |  | — |  |  |  |  |  |  |  |
|  |  | p-value |  | < .001 |  | < .001 |  | — |  |  |  |  |  |  |  |
| Multisensory  Immersion |  | Spearman's rho |  | 0.280 |  | 0.604 |  | 0.457 |  | — |  |  |  |  |  |
|  |  | p-value |  | < .001 |  | < .001 |  | < .001 |  | — |  |  |  |  |  |
| Artistic  Activity |  | Spearman's rho |  | 0.009 |  | -0.060 |  | -0.065 |  | -0.058 |  | — |  |  |  |
|  |  | p-value |  | 0.883 |  | 0.312 |  | 0.274 |  | 0.331 |  | — |  |  |  |
| Nicotine Dependency |  | Spearman's rho |  | -0.180 |  | -0.157 |  | -0.181 |  | -0.168 |  | 0.277 |  | — |  |
|  |  | p-value |  | 0.002 |  | 0.008 |  | 0.002 |  | 0.005 |  | < .001 |  | — |  |
|  | | | | | | | | | | | | | | | |

Note. The entries are rho coefficients. As shown in the table, all factors are negatively correlated with tobacco consumption, except for the last one. The negative correlations for Physical Activity, Social Relationship, Passion and Daily Engagement, and Multisensory Immersion were significant at p < .05.
